# Metabolic Dysfunction-Associated Steatotic Liver Disease in People Living with HIV—Limitations on Antiretroviral Therapy Selection

**DOI:** 10.3390/life14060742

**Published:** 2024-06-10

**Authors:** Georgios Kalopitas, Konstantinos Arvanitakis, Olga Tsachouridou, Konstantinos Malandris, Theocharis Koufakis, Symeon Metallidis, Georgios Germanidis

**Affiliations:** 1 First Department of Internal Medicine, AHEPA University Hospital, Aristotle University of Thessaloniki, 54636 Thessaloniki, Greece; gekalopi@auth.gr (G.K.); arvanitak@auth.gr (K.A.); olgatsachouridou.iasis@gmail.com (O.T.); metallidissimeon@yahoo.gr (S.M.); 2 Basic and Translational Research Unit, Special Unit for Biomedical Research and Education, School of Medicine, Faculty of Health Sciences, Aristotle University of Thessaloniki, 54636 Thessaloniki, Greece; 3 Clinical Research and Evidence-Based Medicine Unit, Second Medical Department, Aristotle University of Thessaloniki, 54124 Thessaloniki, Greece; kostas_malandris@yahoo.gr; 4 2nd Propedeutic Department of Internal Medicine, Hippokration General Hospital, Aristotle University of Thessaloniki, 54642 Thessaloniki, Greece; thkoyfak@auth.gr

**Keywords:** HIV, human immunodeficiency virus, NAFLD, MASLD, ART

## Abstract

Chronic liver disease is one of the main causes of morbidity and mortality in people living with HIV (PLWH). The increasing life expectancy of PLWH, effective treatment for viral hepatitis, and Western dietary patterns as well as the adverse effects of antiretroviral therapy (ART) have rendered metabolic dysfunction-associated steatotic liver disease (MASLD) the most common chronic liver disease in PLWH. The risk factors for MASLD in PLWH include traditional MASLD risk factors and additional virus-specific factors, including the adverse effects of ART. The management of patients suffering from HIV and MASLD is often challenging. Apart from the conventional management of MASLD, there are also certain limitations concerning the use of ART in this patient population. In general, the appropriate combination of antiretroviral drugs should be chosen to achieve the triad of effective viral suppression, avoidance of mitochondrial dysfunction, and deterrence of worsening the patient’s metabolic profile. In the current review, we discuss the epidemiology of MASLD in PLWH, the risk factors, and the disease pathogenesis, as well as the limitations in the use of ART in this patient population, while practical recommendations on how to overcome these limitations are also given.

## 1. Introduction

Non-alcoholic fatty liver disease (NAFLD) and acquired immunodeficiency syndrome (AIDS) are two diseases that have parallel paths. Both were first recognized in the early 1980s and have since then gone hand in hand, as they have significantly affected health systems worldwide.

The term “non-alcoholic steatohepatitis” (NASH) was first introduced in 1980 by Ludwig et al. [1] in a case series of 20 patients who were diagnosed with chronic liver disease of unknown etiology, which histologically resembled alcoholic hepatitis and could gradually progress to advanced fibrosis and cirrhosis in the absence of significant alcohol consumption. Since then, our knowledge regarding this disease has broadened considerably, and NAFLD is currently considered to be a modern liver pandemic, affecting more than 25% of people worldwide, evolving into the most common chronic liver disease in the Western world [2]. Recently, a new nomenclature was given to this disease, aiming to define it more correctly and while also avoiding stigmatization of people who suffer from it [3].

Regarding AIDS, since the first cases were described in the not so distant 1981, human immunodeficiency virus (HIV) and especially AIDS continue to constitute one of the world’s greatest pandemics, despite advances in understanding their pathophysiology and complications, as well as leaps in their therapeutic management [4]. Nowadays, more than 39 million are considered to live with HIV, and more that 1.3 million are believed to have been infected with HIV during the past year [4]. Moreover, 1996 was a landmark year for the disease, when it was proven that triple antiretroviral therapy (ART) could offer effective and long-term suppression of the viral load. The scientific breakthrough of the “triple therapy” is nowadays called highly active antiretroviral therapy (HAART), while the antiretroviral agents that were later introduced have achieved, in addition to the long-term virological suppression, a substantial recovery of the patients’ immune system and thus a reduction in the prevalence of HIV-related diseases, prolonging the overall survival of individuals with HIV [5].

Even though people living with HIV (PLWH) were traditionally considered to be at a greater risk of chronic liver disease and especially non-alcoholic steatohepatitis in the era before the development of HAART, the discovery of HAART did not reduce the risk of non-alcoholic hepatic steatosis, while certain antiretrovirals seem to have even increased it, rendering NAFLD the most common chronic liver disease among PLWH [6]. Moreover, the life expectancy of PLWH under HAART is now approaching that of HIV-uninfected individuals, resulting in PLWH being older and often suffering from other metabolic comorbidities such as type II diabetes mellitus (T2DM), arterial hypertension, and dyslipidemia. The frequent coexistence of these comorbidities, combined with the polypharmacy required to treat them, as well as the increased prevalence of NAFLD in this patient population, often raises concerns in daily clinical practice regarding the drug interactions and adverse effects of ART in PLWH [7,8].

The aim of the present review was to highlight certain concerns and limitations that clinicians may face when dealing with individuals who suffer from HIV alongside non-alcoholic steatotic liver disease and also to provide insight into overcoming these obstacles.

## 2. Metabolic Dysfunction-Associated Steatotic Liver Disease Pathogenesis and the New Fatty Liver Nomenclature

NAFLD is nowadays considered the most common chronic liver disease in the Western world, affecting more than one in four adults [2]. As insulin resistance (IR) is a common pathogenetic feature of both metabolic syndrome (MetS) and NAFLD, with their respective prevalences rising in parallel, NAFLD is considered to be the hepatic constituent of MetS. Based upon this and due to the fact that the term “fatty” was considered potentially stigmatizing, a multisociety Delphi consensus was reached on the new fatty liver disease nomenclature in 2023 [3]. Metabolic dysfunction-associated steatotic liver disease (MASLD), the newer disease term, refers to individuals with hepatic steatosis (diagnosed either by radiology or by liver histology) and with at least one of five cardiometabolic risk factors of MetS, in the absence of significant weekly alcohol consumption, as well as other causes of secondary hepatic steatosis. The five cardiometabolic risk factors include increased body mass index (BMI) (25 kg/m^2^) or increased waist circumference (>94 for men and 80 for women, respectively) or adjusted by ethnicity, increased fasting serum glucose concentrations > 100 mg/dL or abnormal 2 h post-load glucose levels > 140 mg/dL or increased HbA1c > 5.7% or T2DM under antidiabetic therapy, increased blood pressure levels > 130/85 mmHg or patient under antihypertensive therapy, increased plasma triglyceride levels > 150 mg/dL or patient under lipid-lowering therapy, as well as decreased plasma HDL levels (<40 mg/dL for men and <50 mg/dL for women, respectively) or patient under lipid-lowering therapy.

In addition, the new fatty liver disease nomenclature introduced newer disease categories, apart from the classic MASLD and alcoholic liver disease (ALD): metabolic and alcohol-related/associated liver disease (MetALD) as well as cryptogenic steatotic liver disease. MetALD refers to patients suffering from MASLD who consume significant amounts of alcohol (140–350 g/week and 210–420 g/week for women and men, respectively), while the term cryptogenic steatotic liver disease probably refers to the former category of “lean or metabolically healthy NAFLD” [3]. As MASLD has become a modern “metabolic” pandemic with a constantly rising incidence, the new nomenclature aims to improve disease awareness and patient identification, through improved patient categorization and non-stigmatization of people suffering from fatty liver disease.

Regarding the pathogenesis of MASLD, the most widely accepted theory nowadays is the “multiple-hit theory” [9]. According to this theory, lipotoxicity as a “central hit” in combination with further “liver-injuring hits” contributes to the pathogenesis of the disease. The hallmark of NAFLD is intrahepatic fat accumulation, which results from increased fatty acid influx to the liver and reduced intrahepatic fatty acid export and oxidation. Ιncreased dietary fatty acid intake, increased de novo lipogenesis, as well as increased lipolysis in the peripheral adipose tissue due to increased IR contribute to increased intrahepatic fatty acid influx, while mitochondrial dysfunction results in inadequate intrahepatic fatty acid oxidation [10,11,12].

The intrahepatic triglyceride accumulation with the subsequent inability to manage excess energy substrates leads to the formation of toxic lipid species as well as increased production of free reactive oxygen species (ROS) resulting in oxidative stress, secretion of unfolded proteins, and activation of the inflammasome and other inflammatory and apoptotic pathways. All the aforementioned processes lead to the induction of hepatocellular inflammation and injury termed steatohepatitis, which is histologically characterized by the coexistence of steatosis, inflammation, and ballooning of the hepatocytes [9]. Additional “hits” that appear to contribute to the pathogenesis of the disease are genetic polymorphisms, depletion of ATP stores, uric acid toxicity, periodic hypoxia in sleep apnea–hypopnea syndrome, and cytokines produced by the gut microbiome [9,13]. In summary, the pathogenetic risk factors of the disease include, among others, obesity, MetS, T2DM, and other metabolic comorbidities, as well as unhealthy dietary habits and genetic predisposition.

Genetic predisposition seems to play an important role not only in the occurrence of NAFLD/NASH but also in its faster progression to fibrosis stages and the development of HCC. Recent studies have implicated certain genetic factors [14]. A single polymorphism in the PNPLA3 gene (I148M) is the most well-characterized genetic risk factor, with several studies showing that patients with this particular polymorphism are at an increased risk of developing NASH [15]. Obesity in patients with this polymorphism maximizes the risk of NASH, accelerates fibrosis progression, and also increases the risk of HCC [15]. Additional known genetic risk factors are the TM6SF2 gene, the MBOAT7 gene, and the loss-of-function mutation in HSD17B13 [16].

## 3. MASLD in PLWH

### 3.1. Changing Epidemiology of Chronic Liver Disease in PLWH

In the era of antiretroviral therapy, mortality and morbidity among PLWH have significantly decreased, and nowadays, the development of AIDS is rare among people on ART. In contrast, chronic liver disease has increased significantly up to 10-fold as compared to the pre-ART era, being among the leading causes of mortality and morbidity in PLWH, alongside cardiovascular disease (CVD) and non-HIV-related cancers [17]. Chronic hepatopathies in PLWH have been present since the beginning of the HIV pandemic; their etiology, however, has changed significantly over the years due to scientific breakthroughs in HIV and viral hepatitis treatment, alongside the lifestyle changes of PLWH [18].

To start with, the discovery of highly effective antiretroviral agents resulted in a dramatic decrease in the incidence of opportunistic liver infections, rendering co-infection with hepatitis B or C viruses the leading causes of liver-related mortality in this patient population. Later, the initial discovery of nucleoside analogues for hepatitis B, which are now an integral part of ART, as well as the discovery of direct-acting antivirals for hepatitis C (HCV), led to a significant reduction in the prevalence of these infections in PLWH. In addition, the incidence of HIV infection from intravenous drug use has decreased significantly in recent years, which has contributed to the decrease in parenteral transmission of HBV and HCV [17]. It should also be taken into account that the abuse of intravenous drugs, apart from the increased risk of infection with hepatitis and hepato-mimicking viruses, can lead to direct hepatotoxicity and severe chronic liver damage, especially when combined with alcohol abuse.

Regarding steatotic liver disease, in the era before the discovery of antiretroviral therapy, its main causes were opportunistic infections, as well as sarcopenia and malnutrition. With the advent of antiretroviral therapy, hepatic steatosis development was firstly associated with hepatotoxicity due to firs-generation nucleoside/nucleotide reverse transcriptase inhibitors (NRTIs), such as didanosine and stavudine, while, later on, it was related to MetS and subsequently to MASLD. As the burden of viral hepatitis in PLWH continues to considerably decrease and the life expectancy of PLWH approaches that of the general population, there is an increased prevalence of comorbidities associated with advanced age, the Western lifestyle, and increased body weight, such as T2DM, MetS, and, subsequently, steatotic liver disease [18]. Steatotic liver disease (MASLD, MetALD, and ALD) has emerged in the last decade as the most common chronic liver disease in people with HIV monoinfection, with a continuously rising prevalence.

### 3.2. Epidemiology of MASLD in PLWH

MASLD is nowadays considered to be the most common chronic liver disease in PLWH. Notably, recent evidence suggests that the prevalence of MASLD in PLWH is higher as compared to that in the general population. A study by Guaraldi et al. [19] demonstrated that individuals with HIV who were not coinfected with hepatitis viruses and were not alcohol abusers had an estimated NAFLD prevalence with the help of a CT-scan, of approximately 37%. In another study conducted by Crum-Cianflone et al. [20] with 216 consecutive HIV patients of an American military clinic undergoing an abdominal ultrasound, 31% of them were diagnosed with NAFLD. In contrast, a newer study conducted by Lui et al. [21] in an Asian population comparing the prevalence of NAFLD in PLWH and healthy volunteers using MRI did not detect statistically significant differences between them, despite the fact that a higher incidence of MASLD was initially found in PLWH. Finally, a recent meta-analysis by Kalligeros et al. concluded that approximately 33% of PLWH suffer from MASLD, while 12% of PLWH suffer from MASLD with at least moderate fibrosis [22]. The most important epidemiological studies regarding MASLD prevalence in PLWH are summarized in Table 1.

Despite the fact that NAFLD/MASLD prevalence seems to be higher in PLWH in comparison to the general population, the current American Association for the Study of Liver Diseases (AASLD) and European Association for the Study of the Liver (EASL) guidelines do not suggest screening for NAFLD in PLWH [14,26]. On the contrary, the European AIDS clinical society (EACS) suggests screening for NAFLD in PLWH at a higher risk, especially those with increased aminotransferases levels, those suffering from MetS or obesity, and those under medication with class D drugs.

### 3.3. Risk Factors and Pathogenesis of MASLD in PLWH

Regarding the risk factors and pathogenesis of MASLD in PLWH, accumulating evidence suggests that, apart from the traditional risk factors for the development of MASLD, additional virus-specific factors are potential contributors. This was denoted in a recent meta-analysis [22], which correlated the diagnosis of NAFLD in PLWH with the traditional cardiometabolic risk factors, such as hypertension, T2DM, hyperlipidemia, and MetS, as well as with the male gender. Regarding the HIV-specific factors, the proposed mechanisms of HIV-induced liver steatosis include HIV-related lipodystrophy, adverse effects of antiretroviral regimens, proinflammatory microbiota of HIV patients, and direct HIV-induced liver injury [27,28].

Antiretroviral regimens have been implicated in the development of steatosis and liver damage in two individual ways. Firstly, by leading to IR as well as hyperlipidemia and, secondly, by resulting in inadequate intrahepatic fatty acid oxidation owing to mitochondrial dysfunction [29,30]. While first-generation ART regimens and PIs were demonstrated to lead to lipodystrophy and mitochondrial damage, modern ART mostly results in liver steatosis through weight gain and IR. Long-term data from observational studies have shown that the duration of ART seems to play a pivotal role in the long-term progress through the stages of fibrosis, rendering hepatic steatosis and, in some cases, advanced liver fibrosis, as the price of achieving virological suppression [20,21,22,31].

HIV appears to significantly alter the composition of the gut microbiota of PLWH, as well as to affect the integrity of the gut mucosa, resulting in bacterial translocation and, thus, a continuous antigenic stimulation of the host’s immune system. The constant low-grade liver inflammation induced by the dysbiotic microbiota through the gut–liver axis when combined with other liver-damaging factors like steatosis leads to the acceleration of chronic liver injury and to the progression of liver fibrosis. Notably, the altered microbiota of PLWH is characterized by poor gut microbial diversity, which resembles that of individuals suffering from obesity and MetS (decreased Firmicutes and Bacteroidetes and increased Enterobacteriaceae) [32].

Regarding viral-induced liver steatosis, evidence has shown that HIV before the commencement of ART and especially in the stages of immune activation results in hepatic steatosis owing to virus-induced IR and hypertriglyceridemia. However, soon after the commencement of treatment with highly effective combined antiretroviral therapy (cART) and the corresponding virological suppression, these direct viral metabolic effects subside [33,34]. Taking all these into account, and influenced by the new SLD nomenclature, one could say that we might not be talking about MASLD in PLWH, but about HIV-related SLD.

## 4. Limitations of ART in PLWH and MASLD

As already mentioned, HIV and MASLD have had a parallel course over the past 40 years. Moreover, the necessarily administered cART immediately after the diagnosis of HIV infection may, on the one hand, improve the prognosis of PLWH but, on the other, predispose PLWH by various mechanisms to the occurrence of MASLD. It is also well known that the liver is the main site of metabolism of more than half of the prescribed drugs, while hepatotoxicity is the most frequent reason for the early discontinuation of clinical trials due to patient safety issues. In summary, there are several limitations that the clinician treating PLWH and MASLD should be aware of. In the following section of our review, the limitations per category of antiretrovirals will be analyzed (Table 2).

### 4.1. Nucleoside/Nucleotide Reverse Transcriptase Inhibitors

NRTIs are very effective agents in the suppression of viral load, and nowadays, they constitute an integral part of the antiretroviral therapy for PLWH; they are usually administered in pairs alongside a third agent in cART. The long-term use of NRTIs has been associated with the development of non-alcoholic steatohepatitis. This is probably because, apart from their main mechanism of action, i.e., the inhibition of viral reverse transcriptase, NRTIs can simultaneously lead to the inhibition of mitochondrial DNA replication by hindering the action of the enzyme intramitochondrial gamma polymerase. This leads to mitochondrial dysfunction and subsequently to inadequate intrahepatic oxidative phosphorylation, as well as to the overproduction of lactates and reactive oxygen species [35]. The inadequate intrahepatic metabolism of fatty acids leads to excessive intrahepatic triglyceride accumulation (steatosis), one of the hallmarks of the MASLD pathogenesis, which, in combination with other factors, results in liver injury (steatohepatitis) and progresses over time fibrosis. Moreover, NRTIs can further impact the development of liver steatosis by inducing IR, dyslipidemia, and peripheral lipodystrophy due to peripheral fat tissue mitochondria inhibition. Therefore, chronic steatotic liver disease associated with NRTIs shares, to a large extent, the same pathophysiological mechanisms as metabolic dysfunction-associated steatohepatitis [36,37,38,39,40].

Since, in most cases, NRTIs are used in pairs, finding the culprit leading to steatosis and steatohepatitis is often not an easy task. However, first-generation NRTIs, i.e., didanosine, stavudine, zalcitabine, and to a smaller extent zidovudine, are more likely to impact the synthesis of mitochondrial DNA and subsequently lead to steatohepatitis. With the advent of newer drugs of this class, the use of these first-generation NRTIs has been almost abandoned in clinical practice, as these agents have been associated with significant side effects owing to mitochondrial dysfunction, in contrast to the modern NRTIs that rarely result in clinically significant mitochondrial toxicity [41,42]. It is also worth noting that these drugs have also been associated with other side effects such as peripheral neuropathy, lipodystrophy, and episodes of pancreatitis.

Regarding the other drugs of the class that are commonly used and are the mainstay of therapy for PLWH, although they do not lead to advanced liver disease after long-term administration, there are some limitations to their use that clinicians may need to consider. Abacavir may be related to a higher risk of dyslipidemia and cardiovascular events, and its use is recommended to be avoided in patients with established CVD or with multiple risk factors for CVD [43]. As patients with MASLD frequently suffer from CVD or are at an increased risk of cardiovascular events, every patient with MASLD and HIV should be carefully stratified for cardiovascular risk before starting abacavir administration, while the benefits as well as potential risks of its use should be weighed. Regarding the remaining commonly used drugs of the class such as emtricitabine, lamivudine, tenofovir disoproxil fumarate (TDF), and tenofovir alafenamide (TAF), there are no basic restrictions on their use regarding cardiovascular risk, with the only important note that TAF can lead to weight gain and possible worsening of MetS [44].

Patients with MASLD frequently suffer from chronic kidney disease owing to diabetic nephropathy, hypertension, or other causes. TDF seems to be associated with renal impairment and should be used with great caution in this patient population [45]. Moreover, as the life expectancy of PLWH increases thanks to the effectiveness of antiretroviral therapy, individuals are more likely to suffer from osteoporosis alongside MASLD. The use of TDF has also been associated with reduced bone mineral density and osteoporosis; therefore, its use should be avoided in this category of patients, and TAF should be used alternatively [46]. Finally, it is worth mentioning that this class of drugs has few drug interactions as it does not affect the function of the CYP-450 enzymes of the liver, an important point if we consider that patients with MASLD are often in need of multiple drugs for the management of metabolic comorbidities.

### 4.2. Non-Nucleoside Reverse Transcriptase Inhibitors

Non-nucleoside reverse transcriptase inhibitors (NNRTIs) are typically administered alongside a dual combination of NRTIs. Although no specific adverse effects have been identified for this class of drugs regarding steatotic liver disease, there are data implicating efavirenz in the occurrence of liver steatosis. A study including the liver biopsies of patients with HIV/HCV co-infection linked efavirenz use to an increased risk of hepatic steatosis [47]. These data were consistent with another study conducted by Macias et al. in individuals with HIV monoinfection and NAFLD, in whom the replacement of efavirenz by raltegravir (an integrase strand transfer inhibitor characterized by a more favorable course of action) in their regimen resulted in a substantial improvement of liver steatosis [48]. Moreover, efavirenz has been associated with increased transaminase concentrations and cases of drug-induced hepatitis. Subsequently, efavirenz should not be used in patients with advanced liver disease, especially with cirrhosis of Child–Pugh B/C grade, and it should be used with caution in individuals who already suffer from MASLD or are at increased risk of MASLD. Regarding the metabolic profile of the other drugs of this class, doravirine, nevirapine, and rilpivirine excel in comparison to efavirenz in terms of their metabolic profile [49], while etravirine seems to exhibit a neutral metabolic effect in comparison to placebo [50].

### 4.3. Entry Inhibitors

Drugs that act at this part of the life cycle of HIV are not commonly used in clinical practice; however, they may be used in specific complicated cases. It is worth mentioning that maraviroc, a CCRT5 inhibitor, has been linked to hepatotoxicity. Maraviroc clearance is both renal and hepatic, with the current recommendations suggesting dose titration in patients with significant hepatic dysfunction to avert increased plasma concentrations. Regarding MASLD, no association with the occurrence of steatosis and steatohepatitis has been observed, although data are relatively scarce [51].

### 4.4. Integrase Strand Transfer Inhibitors

Raltegravir, the first integrase strand transfer inhibitor (INSTI), was first used in 2008, while elvitegravir, dolutegravir, and bictegravir were introduced later on. This class of drugs is, in the majority of cases, the “preferred third agent” used for the treatment of naïve patients, as it is effective and well tolerated. As drugs in this class do not adversely affect the lipid/metabolic profile compared to efavirenz and PIs, they constitute an attractive choice for individuals suffering from hyperlipidemia or at increased risk of CVD and, consequently, from MASLD [48,52]. However, this class of drugs has been related to weight gain in comparison to other drug classes [53]. Moreover, as patients with MASLD frequently suffer from diabetes or prediabetes, it is noteworthy that dolutegravir has significant drug–drug interactions with metformin. It is also of note that, as elvitegravir inhibits CYP3A, clinicians should always keep in mind potential drug–drug interactions when using this agent.

### 4.5. Protease Inhibitors

PIs were first introduced in 1995, constituting an effective alternative to the anti-HIV quiver. Nowadays, PIs are usually used as a “third agent” along with a combination of two NRTIs, but they can also be used as part of an NRTI-sparing regimen and the co-administration of a boosting agent is often necessary. This class of drugs has been linked to metabolic impairment including hyperlipidemia, hyperglycemia, IR, and lipodystrophy, as well as increased fat accumulation and, therefore, predisposes to the occurrence of hepatic steatosis or the worsening of preexisting hepatic steatosis [29,54]. Increased IR has been a known side effect of the first-generation PIs (ritonavir and indinavir) since the beginning of their use [55]. The underlying pathophysiology of this phenomenon, although still under debate, was attributed to the fact that PIs probably lead to increased concentrations of apolipoprotein B and the defective clearance of circulating triglycerides, resulting in increased concentrations of circulating LDL-cholesterol, VLDL-cholesterol, and triglycerides [56]. Although newer PIs have a milder metabolic impact when used as monotherapy, their combination with boosting agents such as cobicistat or ritonavir results in significant metabolic impairment, in contrast with other ART options [36]. In summary, this category of drugs is still considered “less favorable” in comparison to INSTIs, for individuals suffering from MASLD [57].

## 5. Recommendations for the Optimal Management of PLWH and MASLD

Over the past years, the epidemiology, as well as the phenotype of HIV-positive patients suffering from NAFLD, has significantly changed [17,18]. As their life expectancy and the quality of life increases, PLWH tend to exhibit the same risk factors for the development of MASLD that uninfected patients do. As already mentioned, ART frequently predisposes to metabolic deterioration, increased IR, and the onset or worsening of preexisting hepatic steatosis. In the previous paragraphs, we analyzed the new nomenclature of steatotic liver disease, the epidemiology and etiology of the disease in PLWH, and the effects of different classes of antiretroviral medications on the onset or worsening of steatosis. In the following part of the text, we will discuss the optimal management of PLWH and MASLD, and we will propose ways to overcome the limitations of ART use in this specific patient group.

After the initial diagnosis of an HIV infection, all patients at high risk for MASLD should be screened for steatosis with the help of an upper abdominal ultrasound. The risk factors for MASLD include obesity, T2DM, MetS, hyperlipidemia, arterial hypertension, elevated transaminase concentrations, and D-class drug administration. Once hepatic steatosis is identified, some further steps should be taken to optimally manage these patients. Firstly, the possibility of co-infection with HCV should be investigated, and if confirmed, individuals should be treated with the highly potent direct-acting antivirals of the new generation, since HCV and especially HCV genotype-3 lead to hepatic steatosis [58]. Alcohol abuse and other secondary causes of hepatic steatosis should also be ruled out. If this is the case, the diagnosis of NAFLD (or MASLD if the patient meets at least one of the five required cardiometabolic criteria) is established. All individuals diagnosed with NAFLD/MASLD should be screened using non-invasive liver stiffness tools (FIB4 score or fibroscan) to rule out or diagnose advanced liver fibrosis. In the case of advanced liver fibrosis or cirrhosis, further consultation with a liver specialist should be scheduled [14,27].

Regarding the therapeutic management of PLWH and NAFLD/MASLD, each individual parameter of MetS should be treated separately but also holistically in this group of patients, and the issue of IR should be addressed. For example, patients who are overweight or obese are advised to lose at least 7–10% of their body weight gradually over a year [26] with the help of a hypocaloric healthy diet and increased physical activity. To achieve this goal, the contribution of a nutritionist and frequent exercise counseling seems to be particularly helpful. Patients should also be educated about the harmful effects of alcohol on their condition, and complete abstinence should be suggested. Furthermore, hyperlipidemia and T2DM should be addressed with the help of lifestyle modifications and medications according to clinical practice guidelines, while newer effective pharmaceutical agents will soon be available for the management of MASLD/NAFLD, as resmetirom has recently been officially approved for the treatment of steatohepatitis [59].

However, in addition to the conventional treatment of MASLD/NAFLD, the clinician or the infectious disease specialist who is called to manage an individual who is also suffering from HIV, must always bear in mind some important points, especially regarding the optimal use of ART. First, the risk of mitochondrial toxicity should be minimized by avoiding the use of first-generation NRTIs, such as stavudine and didanosine. Regarding modern NRTIs, as abacavir has been linked to an increased risk of dyslipidemia and CVD, it should be avoided in patients with CVD or those who are at a high risk for CVD [43]. The clinician must also keep in mind that TAF can lead to weight gain and possibly aggravate IR and MetS in patients with increased body weight [44], while TDF should not be used in patients suffering from chronic kidney disease or osteoporosis or in patients older than 65 years, as it has been associated with nephrotoxicity and osteoporosis.

Secondly, the use of a “metabolically friendly” third antiretroviral agent is highly suggested, such as raltegravir, atazanavir, darunavir, or nevirapine. INSTIs are probably the “preferred third agent” for individuals with MASLD, as they are well tolerated, highly efficacious, and have been linked to favorable metabolic effects as compared to the other drug classes [57]. However, physicians must consider that raltegravir leads to weight gain, dolutegravir is related to significant drug–drug interactions with Metformin, and elvitegravir affects CYP3A function. Regarding NNRTIs, nevirapine seems to be more suitable for patients with MASLD, in comparison to efavirenz. As for PIs, although the newer agents atazanavir and darunavir are considered “metabolically neutral” as compared to the first-generation drugs of this class, the necessary co-administration of a boosting agent such as cobicistat or ritonavir renders this ART category a “less favorable” one for individuals suffering from MASLD, when compared with INSTIs [57].

**Figure 1:**
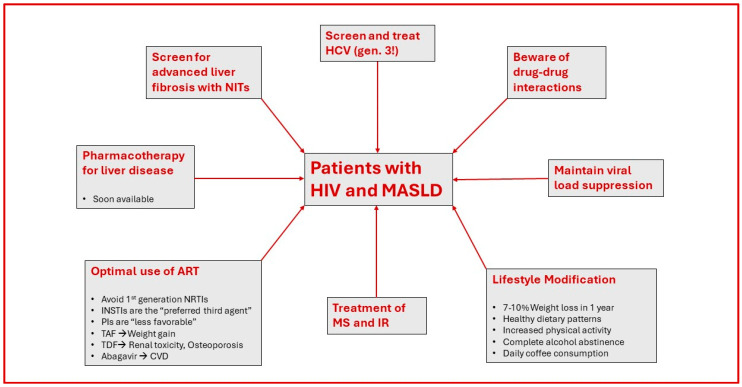
Optimal management of patients with HIV and MASLD. HCV: hepatitis C virus; HIV: human immunodeficiency virus; MASLD: metabolic dysfunction-associated steatotic liver disease; INSTIs: integrase strand transfer inhibitors; IR: insulin resistance; MS: metabolic syndrome; NITs: non-invasive tests; NRTIs: nucleoside/nucleotide reverse transcriptase inhibitors; PIs: protease inhibitors; TAF: tenofovir alafenamide; TDF: tenofovir disoproxil fumarate.

Finally, physicians should never forget that maintaining HIV viral load suppression with potent and “metabolically favorable” antiretroviral agents, averts the prospect of immediate HIV-induced hepatic steatosis and, consequently, leads to the successful treatment of both diseases at the same time. The optimal management of PLWH and MASLD is summarized in Figure 1.

## 6. Conclusions

In conclusion, advances in HIV treatment have led to an increased life expectancy in PLWH. The increasing life expectancy of PLWH, effective treatments for viral hepatitis, and Western lifestyle, as well as the adverse effects of ART have rendered MASLD the most common chronic liver disease. Pathogenetic risk factors for MASLD in PLWH include both the traditional risk factors for MASLD and HIV-specific risk factors including ART-related adverse effects. The management of patients suffering from HIV and MASLD is often challenging as, apart from the conventional management of MASLD, there are also certain limitations regarding the optimal use of ART in this patient population. The goal of optimal ART therapy in this patient population is to effectively suppress the viral load, avert the prospect of mitochondrial toxicity, and at the same time avoid metabolic impairment. The clinicians should be well aware of the limitations of ART and how to overcome them when their patients also suffer from MASLD.

## Figures and Tables

**Table 1:** Main epidemiological studies examining NAFLD/MASLD prevalence in PLWH.

Study ID (First Author, Publication Year, Country)	Total Population (N)	Mean BMI (kg/m^2^)	Diagnostic Method	Prevalence of NAFLD (%)
Guaraldi, 2008, Italy [19]	N = 225	23.8	CT	36.9%
Crum-Cianflone, 2009, USA [20]	N = 216	26	US	31%
Nishijima, 2014, Japan [23]	N = 435	22.8	US	31%
Macias, 2014, Spain [24]	N = 505	23.2	CAP	40%
Vuille-Lessard, 2016, Canada [25]	N = 300	26.6	CAP	48%
Lui, 2016, China [21]	N = 80	23.6	MRS	28.8%

BMI: body mass index; CAP: controlled attenuation parameter; CT: computed tomography scan; MRS: magnetic resonance spectroscopy; NAFLD: non-alcoholic fatty liver disease; US: ultrasound.

**Table 2:** ART limitations in patients with MASLD.

Class of ART	Mechanism of Action	Class-Related Adverse Effects and Limitations	Drug-Related Adverse Effects and Limitations
NRTIs	Inhibit viral reverse transcriptase Simultaneously inhibit host’s mitochondrial DNA replication	Long-term use: liver steatosis, IR 1st generation NRTIs *: mitochondrial toxicity Modern NRTIs: neutral metabolic effects	Abacavir: dyslipidemia, increased risk of CVD TAF: weight gain TDF: nephrotoxicity, osteoporosis
NNRTIs	Inhibit viral reverse transcriptase	No specific class-related effects	Efavirenz: Liver steatosis and DILI Do not use in advanced liver disease
INSTIs	Prevent viral DNA integration into the host cell genome	No specific class-related effects The most “metabolically favorable” drug class	Dolutegravir: drug interactions with metformin Elvitegravir: inhibits CYP3A
PIs	Inhibit viral maturation → Production of immature non-infectious virions	Significant metabolic impairment The least “metabolically favorable” drug class	No specific drug-related limitations
Entry Inhibitors	Inhibition of viral entry into the host cell by attaching to the surface proteins of either CD4 cells or HIV	No specific class-related effects	Maraviroc: hepatic clearance → Dose adjustment in significant hepatic dysfunction

ART: antiretroviral therapy; CVD: cardiovascular disease; DILI: drug-induced liver injury; HIV: human immunodeficiency virus; INSTIs: integrase strand transfer inhibitors; IR: insulin resistance; NAFLD: non-alcoholic fatty liver disease; NRTIs: nucleoside/nucleotide reverse transcriptase inhibitors; NNRTIs: non-nucleoside/nucleotide reverse transcriptase inhibitors; PIs: protease inhibitors; TAF: tenofovir alafenamide; TDF: tenofovir disoproxil fumarate. * First-generation NRTIs: didanosine, stavudine, zalcitabine, and, to a smaller extent, zidovudine.

## Data Availability

No new data were created or analyzed in this study. Data sharing is not applicable to this article.

## References

[1] Ludwig J., Viggiano T.R., McGill D.B., Oh B.J. (1980). Nonalcoholic steatohepatitis: Mayo Clinic experiences with a hitherto unnamed disease. Mayo Clin. Proc..

[2] Younossi Z.M., Golabi P., Paik J.M., Henry A., Van Dongen C., Henry L. (2023). The global epidemiology of nonalcoholic fatty liver disease (NAFLD) and nonalcoholic steatohepatitis (NASH): A systematic review. Hepatology.

[3] Rinella M.E., Lazarus J.V., Ratziu V., Francque S.M., Sanyal A.J., Kanwal F., Romero D., Abdelmalek M.F., Anstee Q.M., Arab J.P. (2023). A multisociety Delphi consensus statement on new fatty liver disease nomenclature. Hepatology.

[4] WHO (2016). Global Health Sector Strategy on HIV 2016–2021. Towards Ending AIDS.

[5] Lange J.M.A., Ananworanich J. (2014). The discovery and development of antiretroviral agents. Antivir. Ther..

[6] Price J.C., Thio C.L. (2010). Liver disease in the HIV-infected individual. Clin. Gastroenterol. Hepatol. Off. Clin. Pract. J. Am. Gastroenterol. Assoc..

[7] Verna E.C. (2017). Non-alcoholic fatty liver disease and non-alcoholic steatohepatitis in patients with HIV. Lancet. Gastroenterol. Hepatol..

[8] Van Welzen B.J., Mudrikova T., El Idrissi A., Hoepelman A.I.M., Arends J.E. (2019). A Review of Non-Alcoholic Fatty Liver Disease in HIV-Infected Patients: The Next Big Thing?. Infect. Dis. Ther..

[9] Friedman S.L., Neuschwander-Tetri B.A., Rinella M., Sanyal A.J. (2018). Mechanisms of NAFLD development and therapeutic strategies. Nat. Med..

[10] Benedict M., Zhang X. (2017). Non-alcoholic fatty liver disease: An expanded review. World J. Hepatol..

[11] Mirmiran P., Amirhamidi Z., Ejtahed H.-S., Bahadoran Z., Azizi F. (2017). Relationship between Diet and Non-Alcoholic Fatty Liver Disease: A Review Article. Iran. J. Public Health.

[12] Bleau C., Karelis A.D., St-Pierre D.H., Lamontagne L. (2015). Crosstalk between intestinal microbiota, adipose tissue and skeletal muscle as an early event in systemic low-grade inflammation and the development of obesity and diabetes. Diabetes Metab. Res. Rev..

[13] Leung C., Rivera L., Furness J.B., Angus P.W. (2016). The role of the gut microbiota in NAFLD. Nat. Rev. Gastroenterol. Hepatol..

[14] Rinella M.E., Neuschwander-Tetri B.A., Siddiqui M.S., Abdelmalek M.F., Caldwell S., Barb D., Kleiner D.E., Loomba R. (2023). AASLD Practice Guidance on the clinical assessment and management of nonalcoholic fatty liver disease. Hepatology.

[15] Valenti L., Al-Serri A., Daly A.K., Galmozzi E., Rametta R., Dongiovanni P., Nobili V., Mozzi E., Roviaro G., Vanni E. (2010). Homozygosity for the patatin-like phospholipase-3/adiponutrin i148m polymorphism influences liver fibrosis in patients with nonalcoholic fatty liver disease. Hepatology.

[16] Dongiovanni P., Petta S., Maglio C., Fracanzani A.L., Pipitone R., Mozzi E., Motta B.M., Kaminska D., Rametta R., Grimaudo S. (2015). Transmembrane 6 superfamily member 2 gene variant disentangles nonalcoholic steatohepatitis from cardiovascular disease. Hepatology.

[17] Bosh K.A., Hall H.I., Eastham L., Daskalakis D.C., Mermin J.H. (2021). Estimated Annual Number of HIV Infections—United States, 1981–2019. MMWR Morb. Mortal. Wkly. Rep..

[18] Navarro J. (2022). HIV and liver disease. AIDS Rev..

[19] Guaraldi G., Squillace N., Stentarelli C., Orlando G., D’Amico R., Ligabue G., Fiocchi F., Zona S., Loria P., Esposito R. (2008). Nonalcoholic fatty liver disease in HIV-infected patients referred to a metabolic clinic: Prevalence, characteristics, and predictors. Clin. Infect. Dis. Off. Publ. Infect. Dis. Soc. Am..

[20] Crum-Cianflone N., Dilay A., Collins G., Asher D., Campin R., Medina S., Goodman Z., Parker R., Lifson A., Capozza T. (2009). Nonalcoholic fatty liver disease among HIV-infected persons. J. Acquir. Immune Defic. Syndr..

[21] Lui G., Wong V.W.-S., Wong G.L.-H., Chu W.C.-W., Wong C.-K., Yung I.M.H., Wong R.Y.K., Yeung S.-L., Yeung D.K.-W., Cheung C.S.K. (2016). Liver fibrosis and fatty liver in Asian HIV-infected patients. Aliment. Pharmacol. Ther..

[22] Kalligeros M., Vassilopoulos A., Shehadeh F., Vassilopoulos S., Lazaridou I., Mylonakis E., Promrat K., Wands J.R. (2023). Prevalence and Characteristics of Nonalcoholic Fatty Liver Disease and Fibrosis in People Living with HIV Monoinfection: A Systematic Review and Meta-Analysis. Clin. Gastroenterol. Hepatol. Off. Clin. Pract. J. Am. Gastroenterol. Assoc..

[23] Nishijima T., Gatanaga H., Shimbo T., Komatsu H., Nozaki Y., Nagata N., Kikuchi Y., Yanase M., Oka S. (2014). Traditional but not HIV-related factors are associated with nonalcoholic fatty liver disease in Asian patients with HIV-1 infection. PLoS ONE.

[24] Macías J., González J., Tural C., Ortega-González E., Pulido F., Rubio R., Cifuentes C., Díaz-Menéndez M., Jou A., Rubio P. (2014). Prevalence and factors associated with liver steatosis as measured by transient elastography with controlled attenuation parameter in HIV-infected patients. AIDS.

[25] Vuille-Lessard É., Lebouché B., Lennox L., Routy J.-P., Costiniuk C.T., Pexos C., Giannakis A., Szabo J., Klein M.B., Sebastiani G. (2016). Nonalcoholic fatty liver disease diagnosed by transient elastography with controlled attenuation parameter in unselected HIV monoinfected patients. AIDS.

[26] Marchesini G., Day C.P., Dufour J.F., Canbay A., Nobili V., Ratziu V., Tilg H., Roden M., Gastaldelli A., Yki-Jarvinen H. (2016). EASL-EASD-EASO Clinical Practice Guidelines for the management of non-alcoholic fatty liver disease. J. Hepatol..

[27] Lemoine M., Assoumou L., De Wit S., Girard P.-M., Valantin M.A., Katlama C., Necsoi C., Campa P., Huefner A.D., Schulze zur Wiesch J. (2019). Diagnostic Accuracy of Noninvasive Markers of Steatosis, NASH, and Liver Fibrosis in HIV-Monoinfected Individuals at Risk of Nonalcoholic Fatty Liver Disease (NAFLD): Results from the ECHAM Study. JAIDS J. Acquir. Immune Defic. Syndr..

[28] Guaraldi G., Maurice J.B., Marzolini C., Monteith K., Milic J., Tsochatzis E., Bhagani S., Morse C.G., Price J.C., Ingiliz P. (2020). New Drugs for NASH and HIV Infection: Great Expectations for a Great Need. Hepatology.

[29] Dekkers C.C., Westerink J., Hoepelman A.I.M., Arends J.E. (2018). Overcoming Obstacles in Lipid-Lowering Therapy in Patients with HIV—A Systematic Review of Current Evidence. AIDS Rev..

[30] Limone P., Biglino A., Valle M., Degioanni M., Paola Servato M., Berardi C., Del Rizzo P., Pellissetto C., Carlo Isaia G. (2003). Insulin resistance in HIV-infected patients: Relationship with pro-inflammatory cytokines released by peripheral leukocytes. J. Infect..

[31] Govender R.D., Hashim M.J., Khan M.A., Mustafa H., Khan G. (2021). Global Epidemiology of HIV/AIDS: A Resurgence in North America and Europe. J. Epidemiol. Glob. Health.

[32] Maurice J.B., Patel A., Scott A.J., Patel K., Thursz M., Lemoine M. (2017). Prevalence and risk factors of nonalcoholic fatty liver disease in HIV-monoinfection. AIDS.

[33] El-Sadr W.M., Mullin C.M., Carr A., Gibert C., Rappoport C., Visnegarwala F., Grunfeld C., Raghavan S.S. (2005). Effects of HIV disease on lipid, glucose and insulin levels: Results from a large antiretroviral-naive cohort. HIV Med..

[34] Da Cunha J., Maselli L.M.F., Stern A.C.B., Spada C., Bydlowski S.P. (2015). Impact of antiretroviral therapy on lipid metabolism of human immunodeficiency virus-infected patients: Old and new drugs. World J. Virol..

[35] Kakuda T.N. (2000). Pharmacology of nucleoside and nucleotide reverse transcriptase inhibitor-induced mitochondrial toxicity. Clin. Ther..

[36] Wei Y., Rector R.S., Thyfault J.P., Ibdah J.A. (2008). Nonalcoholic fatty liver disease and mitochondrial dysfunction. World J. Gastroenterol..

[37] Baril J.-G., Junod P., Leblanc R., Dion H., Therrien R., Laplante F., Falutz J., Côté P., Hébert M.-N., Lalonde R. (2005). HIV-associated lipodystrophy syndrome: A review of clinical aspects. Can. J. Infect. Dis. Med. Microbiol. J. Can. Mal. Infect. Microbiol. Med..

[38] Kakuda T.N., Brundage R.C., Anderson P.L., Fletcher C.V. (1999). Nucleoside reverse transcriptase inhibitor-induced mitochondrial toxicity as an etiology for lipodystrophy. AIDS.

[39] Brinkman K., Smeitink J.A., Romijn J.A., Reiss P. (1999). Mitochondrial toxicity induced by nucleoside-analogue reverse-transcriptase inhibitors is a key factor in the pathogenesis of antiretroviral-therapy-related lipodystrophy. Lancet.

[40] Crane H.M., Grunfeld C., Willig J.H., Mugavero M.J., Van Rompaey S., Moore R., Rodriguez B., Feldman B.J., Lederman M.M., Saag M.S. (2011). Impact of NRTIs on lipid levels among a large HIV-infected cohort initiating antiretroviral therapy in clinical care. AIDS.

[41] Boubaker K., Flepp M., Sudre P., Furrer H., Haensel A., Hirschel B., Boggian K., Chave J.P., Bernasconi E., Egger M. (2001). Hyperlactatemia and antiretroviral therapy: The Swiss HIV Cohort Study. Clin. Infect. Dis. Off. Publ. Infect. Dis. Soc. Am..

[42] Robbins G.K., De Gruttola V., Shafer R.W., Smeaton L.M., Snyder S.W., Pettinelli C., Dubé M.P., Fischl M.A., Pollard R.B., Delapenha R. (2003). Comparison of sequential three-drug regimens as initial therapy for HIV-1 infection. N. Engl. J. Med..

[43] Marcus J.L., Neugebauer R.S., Leyden W.A., Chao C.R., Xu L., Quesenberry C.P.J., Klein D.B., Towner W.J., Horberg M.A., Silverberg M.J. (2016). Use of Abacavir and Risk of Cardiovascular Disease Among HIV-Infected Individuals. J. Acquir. Immune Defic. Syndr..

[44] Schafer J.J., Sassa K.N., O’Connor J.R., Shimada A., Keith S.W., DeSimone J.A. (2019). Changes in Body Mass Index and Atherosclerotic Disease Risk Score after Switching from Tenofovir Disoproxil Fumarate to Tenofovir Alafenamide. Open Forum Infect. Dis..

[45] Zimmermann A.E., Pizzoferrato T., Bedford J., Morris A., Hoffman R., Braden G. (2006). Tenofovir-associated acute and chronic kidney disease: A case of multiple drug interactions. Clin. Infect. Dis. Off. Publ. Infect. Dis. Soc. Am..

[46] Huang J.S., Hughes M.D., Riddler S.A., Haubrich R.H. (2013). Bone mineral density effects of randomized regimen and nucleoside reverse transcriptase inhibitor selection from ACTG A5142. HIV Clin. Trials.

[47] Gwag T., Meng Z., Sui Y., Helsley R.N., Park S.-H., Wang S., Greenberg R.N., Zhou C. (2019). Non-nucleoside reverse transcriptase inhibitor efavirenz activates PXR to induce hypercholesterolemia and hepatic steatosis. J. Hepatol..

[48] Macías J., Mancebo M., Merino D., Téllez F., Montes-Ramírez M.L., Pulido F., Rivero-Juárez A., Raffo M., Pérez-Pérez M., Merchante N. (2017). Changes in Liver Steatosis after Switching from Efavirenz to Raltegravir among Human Immunodeficiency Virus-Infected Patients with Nonalcoholic Fatty Liver Disease. Clin. Infect. Dis. Off. Publ. Infect. Dis. Soc. Am..

[49] Maggi P., Bellacosa C., Carito V., Perilli F., Lillo A., Volpe A., Trillo G., Angiletta D., Regina G., Angarano G. (2011). Cardiovascular risk factors in patients on long-term treatment with nevirapine- or efavirenz-based regimens. J. Antimicrob. Chemother..

[50] Lazzarin A., Campbell T., Clotet B., Johnson M., Katlama C., Moll A., Towner W., Trottier B., Peeters M., Vingerhoets J. (2007). Efficacy and safety of TMC125 (etravirine) in treatment-experienced HIV-1-infected patients in DUET-2: 24-week results from a randomised, double-blind, placebo-controlled trial. Lancet.

[51] MacInnes A., Lazzarin A., Di Perri G., Sierra-Madero J.G., Aberg J., Heera J., Rajicic N., Goodrich J., Mayer H., Valdez H. (2011). Maraviroc can improve lipid profiles in dyslipidemic patients with HIV: Results from the MERIT trial. HIV Clin. Trials.

[52] Quercia R., Roberts J., Martin-Carpenter L., Zala C. (2015). Comparative changes of lipid levels in treatment-naive, HIV-1-infected adults treated with dolutegravir vs. efavirenz, raltegravir, and ritonavir-boosted darunavir-based regimens over 48 weeks. Clin. Drug Investig..

[53] Sax P.E., Erlandson K.M., Lake J.E., Mccomsey G.A., Orkin C., Esser S., Brown T.T., Rockstroh J.K., Wei X., Carter C.C. (2020). Weight Gain Following Initiation of Antiretroviral Therapy: Risk Factors in Randomized Comparative Clinical Trials. Clin. Infect. Dis. Off. Publ. Infect. Dis. Soc. Am..

[54] Den Boer M.A.M., Berbée J.F.P., Reiss P., van der Valk M., Voshol P.J., Kuipers F., Havekes L.M., Rensen P.C.N., Romijn J.A. (2006). Ritonavir impairs lipoprotein lipase-mediated lipolysis and decreases uptake of fatty acids in adipose tissue. Arterioscler. Thromb. Vasc. Biol..

[55] Lee G.A., Rao M., Mulligan K., Lo J.C., Aweeka F., Schwarz J.-M., Schambelan M., Grunfeld C. (2007). Effects of ritonavir and amprenavir on insulin sensitivity in healthy volunteers. AIDS.

[56] Liang J.S., Distler O., Cooper D.A., Jamil H., Deckelbaum R.J., Ginsberg H.N., Sturley S.L. (2001). HIV protease inhibitors protect apolipoprotein B from degradation by the proteasome: A potential mechanism for protease inhibitor-induced hyperlipidemia. Nat. Med..

[57] Tsiodras S., Perelas A., Wanke C., Mantzoros C.S. (2010). The HIV-1/HAART associated metabolic syndrome—Novel adipokines, molecular associations and therapeutic implications. J. Infect..

[58] McGovern B.H. (2011). Hepatic steatosis in HIV/HCV-coinfected patients: Time to reevaluate!. Gastroenterology.

[59] Harrison S.A., Bedossa P., Guy C.D., Schattenberg J.M., Loomba R., Taub R., Labriola D., Moussa S.E., Neff G.W., Rinella M.E. (2024). A Phase 3, Randomized, Controlled Trial of Resmetirom in NASH with Liver Fibrosis. N. Engl. J. Med..

